# Amycolatopsis *aidingensis* sp. nov., a Halotolerant Actinobacterium, Produces New Secondary Metabolites

**DOI:** 10.3389/fmicb.2021.743116

**Published:** 2021-12-06

**Authors:** Rui Li, Meng Wang, Zhen Ren, Yang Ji, Min Yin, Hao Zhou, Shu-Kun Tang

**Affiliations:** ^1^Key Laboratory for Microbial Resources of the Ministry of Education, School of Life Sciences, School of Medicine, School of Chemical Science and Technology, Yunnan Institute of Microbiology, Yunnan University, Kunming, China; ^2^School of Agriculture and Life Sciences, Kunming University, Kunming, China

**Keywords:** halophilic actinomycete, amycoletate, genome sequence, secondary metabolites, *Amycolatopsis aidingensis* sp. nov.

## Abstract

A novel actinobacterium, strain YIM 96748^T^, was isolated from a saline soil sample collected from the south bank of Aiding Lake in Xinjiang Province, Northwest China. Phylogenetic analysis based on 16S rRNA gene sequences revealed that strain YIM 96748^T^ is closely related to *Amycolatopsis cihanbeyliensis* BNT52^T^ (98.9%) and *Amycolatopsis jiangsuensis* KLBMP 1262^T^ (97.2%). The DNA–DNA relatedness between strain YIM 96748^T^ and its closest type strain *A. cihanbeyliensis* BNT52^T^ was 59.6%. The average nucleotide identity between strain YIM 96748^T^ and its neighbor strain was 88.97%. Based on the genotypic and phenotypic characteristics, it is concluded that strain YIM 96748^T^ represents a novel species of the genus *Amycolatopsis*, whose name was proposed as *Amycolatopsis aidingensis* sp. nov. The type strain is YIM 96748^T^. To investigate the biosynthetic potential of producing secondary metabolites, the complete genome of YIM 96748^T^ was sequenced and analyzed. The complete genome sequence of YIM 96748^T^ consists of a 7,657,695-bp circular chromosome, comprising 7,162 predicted genes with a DNA G + C content of 70.21 mol%. Fifty-one putative biosynthetic gene clusters of secondary metabolites were found, including the antibacterial/antitumor agent TLN-05220, the antibacterial agent nocardicin A, the antifungal agent nystatin A1, and the osmolyte ectoine. The investigation of the secondary metabolites of *A. aidingensis* YIM96748^T^ led to the discovery of two new phenylpropyl acetate enantiomers, amycoletates A (1) and B (2), and five known compounds: 4-hydroxy phenethyl acetate (3), 2-*p*-acetoxyphenylethanol (4), (*S*)-ethyl indole-3-lactate (5), (*R*)-ethyl indole-3-lactate (6), and *p*-hydroxybenzoic acid (7). One of the gene clusters 14, 36, and 43, which contain a single module of polyketide synthase, might be responsible for the biosynthesis of compounds 1 and 2 from compound 7 as a precursor. Further studies, including the one strain many compounds approach (OSMAC) and genetic modification, are needed to explore novel compounds from this talented halophilic *Amycolatopsis* strain.

## Introduction

Strains of the genus *Amycolatopsis* are valuable sources, which could produce many active secondary metabolites. Glycopeptide antibiotic vancomycin from *Amycolatopsis orientalis* has been used to treat bacterial infections, including methicillin-resistant *Staphylococcus aureus* (MRSA), for decades (Foldes et al., 1983). The ansamycin antibiotic rifamycin from *Amycolatopsis mediterranei* S699 is one of the first-line therapies used for the treatment of pathogenic Gram-positive cocci and mycobacteria infections for more than half a century (Sepkowitz et al., 1995). Besides, many powerful antibiotics, such as barrymycin, balhimycin, chloroeremomycin, telavancin, oritavancin, and dethymicin, have been discovered from this genus. The earliest discovered species of *Amycolatopsis* is *Streptomyces orientalis* (Brigham and Pittenger, 1956). It was renamed as *Amycolatopsis orientalis* in 1986 (Lechevalier et al., 1986). Eighty-eight species have been validly published with the correct name in this genus until now. Members of the genus *Amycolatopsis* are aerobic or facultative aerobic, Gram-positive, catalase-positive, non-acid-fast, non-motile actinomycetes that contain *meso*-diaminopimelic acid in the wall peptidoglycan (Lechevalier and Lechevalier, 1970). Fatty acids are rich in *iso*- and *anteiso*-branched components and show a lack of mycolic acids (Takahashi, 2001). The predominant menaquinone type is MK-9(H_4_), and the G + C content of the genomic DNA ranges from 66 to 75 mol%.

Halophilic actinomycete strains are regarded as one of the valuable resources for the discovery of useful metabolites, such as antibiotics, compatible solutes, and potential industrial useful polymers. During our research on the resources of actinomycetes in the high-salt environment in Xinjiang Province, a novel actinobacterial strain, YIM 96748^T^, with siderophore activity was isolated from the south bank of Aiding Lake, which is situated in the southern part of the Turpan Basin in Xinjiang Province, Northwest China. In this study, YIM 96748^T^ was recognized as a novel species of the genus *Amycolatopsis* through a polyphasic approach, and its name was proposed as *Amycolatopsis aidingensis* sp. nov. Seven metabolites, including two new ones, were obtained from fermentation extracts. The complete genome of this talented halophilic actinomycete strain was sequenced and analyzed. Putative secondary metabolite biosynthetic gene clusters were investigated to guide the discovery of novel natural products.

## Materials and Methods

### Bacterial Isolation

Strain YIM 96748^T^ was isolated on cellulose–casein multi-salt (CCMS) medium (Tang et al., 2008) supplemented with 5% NaCl (*w*/*v*), which had been inoculated with a soil suspension and incubated at 37°C for 4 weeks from soil samples isolated from a saline soil sample collected from the south bank of Aiding Lake, which is situated in the southern part of the Turpan Basin in Xinjiang Province, Northwest China. The isolate was purified on and maintained on yeast extract–malt extract agar (ISP 2) and preserved as a suspension of mycelial fragments in glycerol (20%, *v*/*v*) at −80°C.

### 16S Ribosomal RNA Gene Sequence, Phylogenetic Analysis, and G + C Content

DNA isolation, 16S ribosomal RNA (rRNA) gene amplification, and sequencing were done as described by Feng et al. (2020). Identification of the phylogenetic neighbors and the calculation of pairwise 16S rRNA gene sequence identities were achieved using the EzTaxon-e database^1^ (Yoon et al., 2017). Phylogenetic analyses were carried out using three treeing algorithms—the neighbor-joining (Saitou and Nei, 1987), maximum likelihood (Felsenstein, 2005), and maximum parsimony (Fitch, 1971) methods—with MEGA version 7.0 (Kumar et al., 2016) and bootstrap values based on 1,000 replications (Felsenstein, 1985). The DNA G + C mole percent value was obtained from the genomic sequence. DNA–DNA hybridization (DDH) was determined using the *in silico* method with the Genome-to-Genome Distance Calculator server version 2.1 (Meier-Kolthoff et al., 2012). The average nucleotide identity (ANI) was calculated with OrthoANI (Richter et al., 2016). The average amino acid identity (AAI) values were calculated from the protein sequences using an online AAI calculator.^2^ Protein sequences were predicted from the genome sequences using GeneMarkS (Besemer et al., 2001). The strain *Amycolatopsis cihanbeyliensis* BNT52^T^ was used as the reference strain in the ANI value calculation and digital DDH (Stamatakis, 2014).

### Physiological, Morphological, and Biochemical Tests

The cultural and morphological characteristics of the isolate were determined after growth for 7 and 14 days at 37°C on the following media: ISP 2, ISP 3, ISP 4, and ISP 5, Czapek’s agar, potato dextrose agar (PDA), and nutrient agar (NA) media with 5% (*w*/*v*) NaCl concentration. Cell morphology was examined using a light microscope (DM2000; Leica, Wetzlar, Germany) and a scanning electron microscope (XL30 ESEM-TMP; Philips-FEI, Eindhoven, Netherlands), with cells grown on ISP 2 medium for 14 days at 37°C. Growth was tested at different temperatures (4, 10, 15, 20, 25, 28, 30, 35, 37, 40, 45, 50, and 55°C) and at pH 4.0–12.0 (at intervals of 0.5 pH unit) using the buffer system described by Xu et al. (2005) and for NaCl tolerance (0–30%, *w*/*v*) using the ISP 2 medium after 14 days of incubation at 37°C. Carbon source tests for growth were carried out on ISP 9 containing 5% (*w/v*) NaCl, as described by Shirling and Gottlieb (1966). Nitrogen source utilization tests were carried out as described by Gordon et al. (1974). Catalase activity was determined by bubble production in 3% (*v*/*v*) H_2_O_2_, and oxidase activity was determined using 1% (*w*/*v*) oxidation of tetramethyl-*p*-phenylenediamine. Gelatinase activities, starch hydrolysis, nitrate reduction, urease, milk peptonization and coagulation, and H_2_S and melanin production were assessed as described by Smibert and Krieg (1994). The other enzyme activities were determined using the API ZYM and API 20NE systems (bioMérieux, Marcy l’Etoile, France) according to the manufacturer’s instructions.

### Biochemical Characteristics

Biomass for quantitative fatty acid analysis was obtained from cultures grown in tryptic soy broth (TSB) for 7 days at 37°C and 200 rpm. Biomass for other chemotaxonomic studies was obtained after cultivation at 37°C for 7 days in shaken cultures with ISP 2 containing 5% (*w*/*v*) NaCl. The whole-cell sugar pattern and peptidoglycan amino acids were identified by high-performance liquid chromatography (HPLC) according to the methods used by Tang et al. (2009). The isomer of diaminopimelic acid in whole-cell hydrolyzates was determined using thin-layer chromatography (TLC) as described by Lechevalier and Lechevalier (1970) and Hasegawa et al. (1983). Polar lipids were extracted, examined by two-dimensional TLC, and identified using previously described procedures (Minnikin et al., 1984). Menaquinones were extracted and purified as described by Collins et al. (1977) and analyzed using HPLC (Groth et al., 1996). Cellular fatty acid analysis was performed using the microbial identification system (Sherlock version 6.1; MIDI database: TSBA6).

### Genome Sequencing and Mining

A single colony of YIM 96748^T^ was inoculated into 50 ml TSB liquid (BD Biosciences, Franklin Lakes, NJ, United States) for 40 h at 28°C with 250 rpm vigorous shaking. DNA isolation and purification in YIM 96748^T^ were carried out according to standard procedures (Pospiech and Neumann, 1995). The quality and quantity of purified genomic DNA were analyzed using the NanoDrop 2000 spectrophotometer (Thermo Fisher Scientific, Waltham, MA, United States) and 0.8% agarose gel electrophoresis.

Genomic DNA was sheared to 8–10-kb length fragments randomly and a genomic DNA library constructed for PacBio sequencing. PacBio clean data were generated by the sequencing platform, and all reads were assembled and checked using HGAP software (Rhoads and Au, 2015). The protein coding sequences were predicted with Glimmer (v3.02) on NCBI (Delcher et al., 2007), and the gene functions were annotated using the NCBI Prokaryotic Genome Annotation Pipeline. The Clusters of Orthologous Groups of proteins (COG) and Gene Ontology (GO) programs (Tatusov et al., 2003; The Gene Ontology Consortium, 2015) were used to analyze the function of the annotated genes. Kyoto Encyclopedia of Genes and Genomes (KEGG) pathway analysis (Kanehisa and Goto, 2000) was carried out to determine the key potential pathways in YIM 96748^T^.

The CRISPR finder platform (Grissa et al., 2007) was used to identify the CRISPR-Cas sequences on the chromosome. ResFinder (Zankari et al., 2012) and the Comprehensive Antibiotic Resistance Database (CARD) (McArthur et al., 2013) were used to predict resistance genes. The VFDB database (Chen et al., 2016) was used to predict the bacterial virulence factors, and PHAST (Zhou et al., 2011) was used to identify putative prophages on the chromosome. The biosynthetic gene clusters for putative secondary metabolites were identified using the antiSMASH 5.0 program (Blin et al., 2019) and verified by manual inspection.

### Fermentation

Strain YIM96748^T^ was activated in ISP 2 medium (4 g soytone, 10 g malt extract, 4 g glucose, 1,000 ml H_2_O, pH 7.1–7.2) at 37°C for 5 days. The activated strain was inoculated into 500-ml Erlenmeyer flasks containing 100 ml of the ISP 2 liquid medium and cultured for 7 days at 37°C and 200 rpm. Then, 5.0 ml of the seed cultures were transferred into 500-ml Erlenmeyer flasks containing 100 ml of fermentation medium [20 ml glycerol, 20 g dextrin, 10 g soytone, 2 g yeast extract, 2 g (NH_4_)_2_SO_4_, 2 g CaCO_3_, 1,000 ml H_2_O, pH 7.0] and cultured for 10 days at 37°C and 200 rpm (total, 10 L).

### Isolation and Identification of Compounds

The broth was extracted with 10 L ethyl acetate (EtOAc) three times, and the solvent was removed under vacuum to obtain the EtOAc extract (5.12 g). The crude extract was separated into five fractions (Fr.1–Fr.5) with column chromatography (CC) on silica gel, eluting stepwise with a CHCl_3_–MeOH gradient [CHCl_3_, CHCl_3_–MeOH = 30:1 (*v*/*v*), CHCl_3_–MeOH = 10:1 (*v*/*v*), CHCl_3_–MeOH = 1:1 (*v*/*v*), MeOH]. Fr.2 was divided into three parts (Fr.2-1–Fr.2-3) with a silica gel column with petroleum ether–ethyl acetate (50:1–1:1, *v*/*v*), and then Fr.2-2 was separated by a Sephadex LH-20 column with MeOH to obtain compound 3 (8.9 mg). Fr.2-3 was fractionated by a silica gel column with dichloromethane–ethyl acetate (50:1–1:1, *v*/*v*) to afford compounds 5 (1.0 mg) and 6 (1.2 mg). Fr.3 was divided into three parts (Fr.3-1–Fr.3-3) by a silica gel column with dichloromethane–ethyl acetate (80:–1:1, *v*/*v*). Then, Fr.3-1 was separated using preparative HPLC to yield compound 7 (10.2 mg). Fr.3-2 was fractionated using a Sephadex LH-20 column with MeOH to obtain compounds 1/2 (0.9 mg) and 4 (3.4 mg).

NMR spectra, including 1D and 2D spectra, were acquired at room temperature on a Bruker Avance 400-MHz instrument (Bruker, Karlsruhe, Germany). High-resolution electrospray ionization mass spectroscopy (HRESIMS) data were obtained on an Agilent G3250AA (Agilent, Santa Clara, CA, United States). Reversed-phase HPLC was performed on an Agilent Eclipse XDB-C_18_ (5 μm) column. Silica gel (200–300 mesh; Qingdao Marine Chemical Group Co., Qingdao, China), Lichroprep RP-18 gel (40–63 mm; Merck, Darmstadt, Germany), and Sephadex LH-20 (GE Healthcare Bio-Science AB, Uppsala, Sweden) were used for CC. Analytical TLC was carried out using HSGF 254 plates (Qingdao Marine Chemical Group Co., Qingdao, China) and visualized by spraying with an anisaldehyde–H_2_SO_4_ reagent.

## Results and Discussion

### Molecular Phylogenetic Analysis

**FIGURE 1:**
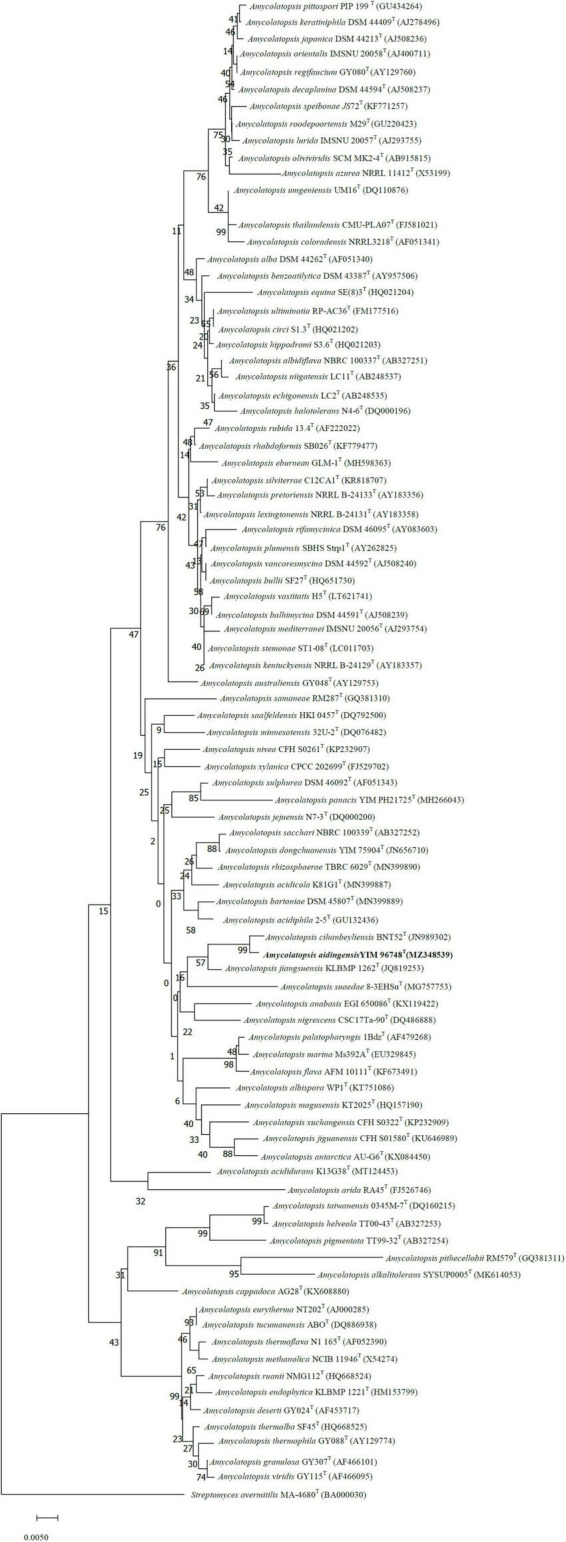
Neighbor-joining tree (MEGA 7.0) showing the relationship between strain YIM 96748^T^ and related taxa. *Streptomyces avermitilis* MA-4680^T^ was used as the outgroup.

The sequence of the 16S rRNA gene of strain YIM 96748^T^ (1,517 bp, accession no. MZ348539) was used for phylogenetic analysis. Phylogenetic analyses of the 16S rRNA gene sequences showed that strain YIM 96748^T^ was a member of the genus *Amycolatopsis*. It is evident from the neighbor-joining tree (Figure 1) that strain YIM 96748^T^ formed a cluster with *A. cihanbeyliensis* BNT52^T^, *Amycolatopsis jiangsuensis* KLBMP 1262^T^, and *Amycolatopsis suaedae* 8-3EHSu^T^ within members of the genus *Amycolatopsis*. The isolate showed the highest 16S rRNA gene sequence similarity with *A. cihanbeyliensis* BNT52^T^ (98.9%). The genome tree demonstrated that YIM 96748^T^ is steadily clustered in a branch with strain *A. cihanbeyliensis* BNT52^T^ (GCA_006715045.1) under the 100 bootstrap values (Supplementary Figure 1). However, the level of DNA–DNA relatedness between strains YIM 96748^T^ and *A. cihanbeyliensis* BNT52^T^ (GenBank accession no. GCA_006715045.1) was only 59.60%, a value well below the 70% threshold recommended for the delineation of bacterial species by Wayne (1988). The ANI value between these two strains was 88.97%. Two-way AAI analysis was used. Strain YIM 96748^T^ and the closely related strain *A. cihanbeyliensis* BNT52^T^ had an AAI value of 88.9%, lower than the 95–96% threshold for species demarcation, confirming that strain YIM 96748^T^ represents a novel species within the genus *Amycolatopsis*. The G + C content of the genomic DNA of the type strain was 70.21 mol%.

### Physiological, Morphological, and Biochemical Tests

**FIGURE 2:**
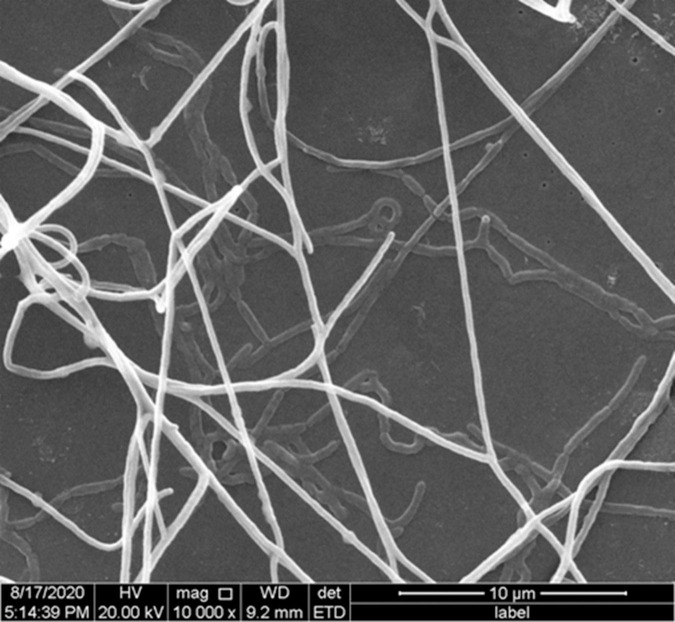
Scanning electron micrograph of the aerial mycelium and substrate mycelium of strain YIM 96748^T^ on ISP 2 with 5% (*w*/*v*) NaCl concentration after incubation for14 days. *Bar*, 10 μm.

The cells of strain YIM 96748^T^ were Gram-reaction-positive, aerobic, non-motile, catalase-positive actinomycetes, which formed a septal substrate mycelium that fragmented into rod-like elements. Irregular swelling appeared in the part of the aerial mycelium (Figure 2) when grown on ISP 2 media with 5% (*w/v*) NaCl for 14 days. Strain YIM 96748^T^ grew well on ISP 2, ISP 3, ISP 4, and ISP 5 agar, Czapek’s agar, NA, and PDA media. The color of the aerial mycelium was white on ISP 2, ISP 3, ISP 4, and ISP 5 agar, Czapek’s agar, NA, and PDA media. The color of the substrate mycelium was purple on PDA, NA, and ISP 2 media. Diffusible pigments were not produced. The growth of strain YIM 96748^T^ was observed at pH 5–12 (optimum, 8) and in the presence of 1–15% (*w*/*v*) NaCl. The temperature range for growth was 20–45°C, with optimum growth at 37°C. The strain was positive for nitrate reduction, gelatin, milk peptonization, and *para*-nitro-β-D-methyl galactose. It was negative for urease, indole, H_2_S, and hydrolysis of aesculin and starch. Tweens 20, 40, and 80 were hydrolyzed. In the API 20NE test, the strain was positive for urease, malic acid, and maltose. In the API ZYM test, it was positive for alkaline phosphatase, esterase C4, esterase lipase C8, lipase C14, leucine arylamidase, valine arylamidase, cystine arylamidase, trypsin, α-chymotrypsin, α-glucosidase, and *N*-acetyl-β-glucosaminidase. In the API 20NE test, there was assimilation of glucose, arabinose, mannose, mannitol, *N*-acetyl-glucosamine, maltose, gluconate, adipic acid, and citric acid. The physiological properties that distinguished strain YIM 96748^T^ from closely related species of the genus *Amycolatopsis* are listed in Table 1.

**TABLE 1:** Differentiation of the physiological characteristics of strain YIM 96748^T^, *Amycolatopsis cihanbeyliensis* BNT52^T^, *Amycolatopsis jiangsuensis* KLBMP 1262^T^, and *Amycolatopsis suaedae* 8-3EHSu^T^.

Characteristics	1	2	3	4
Growth on ISP 4 medium	Good	Good	Poor	Good
Growth on ISP 3 medium	Good	Good	Moderate	Moderate
Growth on ISP 5 medium	Good	Good	Good	Moderate
Temperature range (°C)	20–45	20–37	15–37	15–45
Optimum temperature (°C)	30–45	28	28	28–37
NaCl range (%, *w*/*v*)	0–15	0–10	0–10	0–11
pH range	5–12	6–12	6–11	6–10
Utilization of:				
D-Arabinose	−	+	−	−
D-Mannitol	+	+	−	−
α-Lactose	+	+	−	+
L-Arabinose	−	−	+	+
Decomposition of:				
Nitrate reduction	+	+	−	+
Aesculin	−	+	NR	+
Gelatin	+	NR	−	+
Tween 20	+	NR	+	−
API ZYM results:		+^a^		−
Cystine arylamidase	+	+^a^	−^a^	−
Trypsin	+	+^a^	−^a^	−
α-Chymotrypsin	−	+^a^	−^a^	−
Acid phosphatase	−	+^a^	+^a^	−
Naphthol-AS-BI-phosphohydrolase	−	+^a^	+^a^	+
β-Galactosidase	−	−^a^	+^a^	−
α-Mannosidase	−	−^a^	+^a^	−
API 20NE results:				
*p*-Nitro-β-D methylgalactose	+	−^a^	+^a^	NR
Assimilation of:				
Glucose	−	+^a^	+^a^	NR
Arabinose	−	+^a^	−^a^	NR
Mannose	−	+^a^	+^a^	NR
Mannitol	−	+^a^	+^a^	NR
*N*-acetyl-glucosamine	−	+^a^	+^a^	NR
Maltose	+	−^a^	−^a^	NR
Gluconate	−	+^a^	+^a^	NR
Adipic acid	−	+^a^	+^a^	NR
Citric acid	−	−^a^	+^a^	NR

*Strains: (1) YIM 96748^T^; (2) A. cihanbeyliensis BNT52^T^ (Tatar et al., 2013); (3) A. jiangsuensis K LBMP 1262^T^ (Xing et al., 2013); (4) A. suaedae 8-3EHSu^T^ (Chantavorakit et al., 2019). +, positive; -, negative. NR, not reported.*

*^a^Date from this study.*

### Biochemical Characteristics

The cell wall diamino acid in the peptidoglycan of strain YIM 96748^T^ was determined to be *meso*-diaminopimelic acid, and the whole-cell sugars include manose (6.8%), ribose (5.3%), glucose (57.8%), and galactose (19.8%) as the major components. The polar lipids were found to include diphosphatidylglycerol, unidentified polar lipids, phosphatidylinositol, phosphatidylethanolamine, unidentified phospholipids, an unidentified amino lipid, and phosphatidylmonomethylethanolamine (Supplementary Figure 2). MK-9(H_4_) was detected as the major menaquinone. The major fatty acids were determined to be iso-C_16:0_ (54.8%) and C_17:1_ *w*6c (15.6%), with C_16:0_ (2.8%), C_18:0_ (2.7%), anteiso-C_17:0_ (2.6%), iso-C_14:0_ (2.3%), iso-C_15:0_ (2.8%), Summed Feature 3 (16:1 *w*6c/16:1 *w*7c) (1.8%), iso-C_16:1_ H (1.7%), C_17:1_ *w*8c (1.6%), C_19:0_ 10-methyl (1.1%), and iso-C_17:0_ (1.1%) present as minor components. The major fatty acid is the same as that of *A. cihanbeyliensis* BNT52^T^, but its content is really different. The chemotaxonomic characteristics of strain YIM 96748^T^ are therefore considered to be typical of members of the genus *Amycolatopsis*. Further detailed biochemical characteristics are listed in Table 2.

**TABLE 2:** Differentiation of the chemotaxonomic characteristics between strain YIM 96748^T^, *Amycolatopsis cihanbeyliensis* BNT52^T^, *Amycolatopsis jiangsuensis* KLBMP 1262^T^, and *Amycolatopsis suaedae* 8-3EHSu^T^.

Characteristics	1	2	3	4
**Major fatty acids (%)**				
*Iso*-C_14:0_	2.3	–	4.6	3.0
*Iso*-C_15:0_	2.8	2.8^a^	12.0	12.2
*Iso*-C_16:0_	54.8	28.5^a^	14.6	16.4
*Iso*-C_16:1_ H	1.7	5.9^a^	NR	NR
*Iso*-C_17:0_	1.1	4.5^a^	3.1	3.3
C_17:1_ *w*8c	1.6	3.5^a^	NR	2.0
C_17:1_ *w*6c	15.6	15.2^a^	NR	NR
*Anteiso*-C_17:0_	2.6	4.6^a^	3	5.2
C_18:0_	2.7	–	2	3.3
C_16:1_ *w*6c/C_16:1_ *w*7c (Summed Feature 3)	1.8	4.6^a^	6.6	10.8
Major polar lipids	DPG, L, PI, PE, PL, AL, PME	PME, DPG, L, PI, PE, PL, AL	PME, L, PE, APL, GL	DPG, PE, OH-PE, AL, L
Diaminopimelic acids	*Meso*-DAP	*Meso*-DAP	*Meso*-DAP	*Meso*-DAP
Whole-cell sugars	Manose, ribose, glucose, galactose	Arabinose, galactose, glucose	Arabinose, galactose	Arabinose, galactose, mannose
DNA G + C (%)	70.2	70.1	70.4	71.8

*Strains: (1) YIM 96748^T^; (2) A. cihanbeyliensis BNT52^T^ (Tatar et al., 2013); (3) A. jiangsuensis K LBMP 1262^T^ (Xing et al., 2013); and (4) A. suaedae 8-3EHSu^T^ (Chantavorakit et al., 2019).. –, not present or is a minor component. DPG, diphosphatidylglycerol; L, unidentified polar lipids; PI, phosphatidylinositol; PE, phosphatidylethanolamine; PL, unidentified phospholipids; AL, unidentified amino lipid; PME, phosphatidylmonomethylethanolamine; GL, unknown glycolipid; APL, unknown aminophospholipid; OH-PE, hydroxyphosphatidylethanolamine; NR, not reported.*

*^a^Date from this study.*

### Description of *Amycolatopsis aidingensis* sp. nov.

*Amycolatopsis aidingensis* (ai.ding.en’sis), N.L. fem. adj. *aidingensis* of or belonging to Aiding Lake, a salt lake in China (where the type strain was isolated). This strain is a Gram-positive, aerobic, non-motile actinobacterium that formed a septal substrate mycelium that fragmented into rod-like elements, with irregular swelling appearing in the part of the aerial mycelium. It grows well on ISP 2, ISP 3, ISP 4, and ISP 5 agar, Czapek’s agar, NA, and PDA media. The color of the aerial mycelium was white on ISP 2, ISP 3, ISP 4, and ISP 5 agar, Czapek’s agar, NA, and PDA media. The color of the substrate mycelium was purple on PDA, NA, and ISP 2 media. Growth occurs at pH 5–12 (optimum, pH 8) at 20–45°C (optimum, 37°C) and with 1–15% (*w*/*v*) NaCl tolerance (optimum, 5% NaCl). D-Trehalose, mannitol, xylitol, D-cellulose, D-mannitol, D-sorbitol, raffinose, D-xylose, maltose, α-lactose, fructose, sodium citrate, amber acid, and α-D-glucose were utilized as sole carbon and energy sources, but not D-galactose, melezitose, oligofructose, L-arabinose, α-methyl glucoside, D-salicylin, β-cyclodextrin, or maltitol. It utilizes xanthine, L-phenylalanine, glycine, L-threonine, L-lysine, L-tyrosine, glutamine, L-asparagine, alanine but not adenine, L-methionine, aspartic acid, arginine, or hypoxanthine as sole nitrogen sources.

The cell wall contained *meso*-diaminopimelic acid, and the predominant menaquinone was MK-9 (H_4_). The major phospholipids consisted of diphosphatidylglycerol, unidentified polar lipids, phosphatidylinositol, phosphatidylethanolamine, unidentified phospholipids, an unidentified amino lipid, and phosphatidylmonomethylethanolamine. The major fatty acids (>10.0%) were iso-C_16:0_ (54.8%) and C_17:1_ *w*6c (15.6%). The DNA G + C content of the type strain is 70.2 mol%. The type strain is YIM 96748^T^ (= KCTC 49720^T^ = CGMCC 4.7734^T^ = CCTCC AA 2021025^T^), isolated from Turpan City, Xinjiang Province, Northwest China.

### Genome Sequencing and Analysis

**FIGURE 3:**
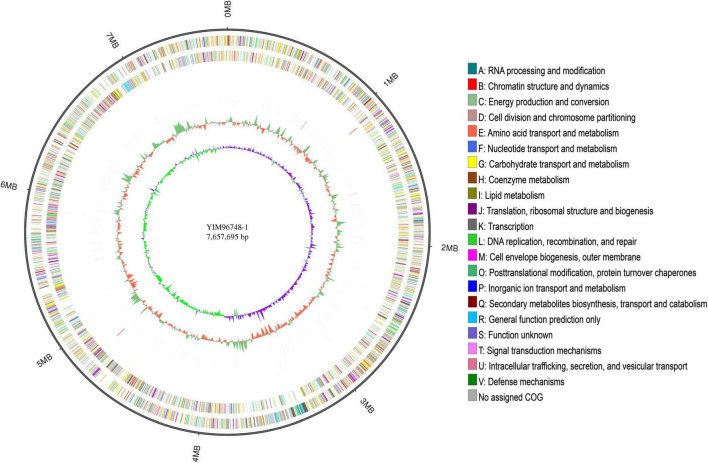
Circular genome map of YIM 96748^T^. Tracks (from *inner* to *outer*): *1*, Genome size; *2*, forward strand gene, colored according to Clusters of Orthologous Groups of proteins (COG) classification; *3*, reverse strand gene, colored according to COG classification; *4*, forward strand ncRNA; *5*, reverse strand ncRNA; *6*, repeat; *7*, GC content; and *8*, GC skew.

The complete genome of YIM 96748^T^ was obtained using the PacBio RSII platform. Approximately 714 Mb PacBio clean data were generated. The average depth of genome coverage was 93-fold. The complete genome of YIM 96748^T^ was composed of a circular chromosome of 7,657,695 bp with a GC content of 70.21 mol% (accession no. GCA_018885265.1). The chromosome contained 7,162 predicted genes, including 6 rRNA genes, 49 transfer RNA (tRNA) genes, and 32 small RNA (sRNA) genes (Figure 3 and Table 3). Among the identified genes, 5,091 and 4,080 were classified into functional categories based on COG and GO designation, respectively (Supplementary Figures 6, 7). Of the KEGG pathways, 3,327 genes were assigned (Supplementary Figure 8). The CRISPR-Cas systems are used by bacteria for defense against the invasion of foreign genetic elements such as viruses and alien plasmids (Barrangou et al., 2007). Twelve putative CRISPR repeat regions were identified on the chromosome of YIM 96748^T^. The length of CRISPR ranged from 89 to 518 bp, and the number of spacers ranged from 1 to 8 (Table 3). One hundred eleven putative antibiotic resistance genes and 377 putative virulence factors were found (Table 3). Five incomplete prophage remnants were detected on the chromosome, and the length of the prophages ranged from 8,264 to 28,169 bp (Table 3).

**TABLE 3:** Genomic features of YIM 96748^T^.

Feature	Chromosome characteristics
Genome topology	Circular
Chromosome size (bp)	7,657,695
GC content (%)	68.78%
Predicted genes	7,162
rRNA operons	6
tRNA genes	79
ncRNA genes	32
Genes assigned to COG	5,091
Genes assigned to GO	4,080
Genes assigned to KEGG	3,327
CRISPR repeat regions	12
Antibiotic resistance genes	111
Virulence factors	377
Prophages	5
Secondary metabolite gene clusters	51

*COG, Clusters of Orthologous Groups of proteins; GO, Gene Ontology; KEGG, Kyoto Encyclopedia of Genes and Genomes.*

### Genome Mining of Secondary Metabolites

A large number of halophilic actinomycetes were isolated in our previous work. Most strains of this group grow very slowly, and little product could be isolated under traditional fermentation conditions. To evaluate the ability of these strains to produce secondary metabolites, the genomes of more than 40 strains were sequenced. Most of them contained very few gene clusters. Among these strains, YIM 96748^T^ contained significantly more gene clusters than did the other strains. Fifty-one putative biosynthetic gene clusters were found (Supplementary Figure 9), including 14 saccharides, 6 polyketides, 6 polyketide–non-ribosomal peptides, 6 non-ribosomal peptides, 3 terpenes, 3 fatty acids, 3 lantipeptides, 2 lasso peptides, 2 NAPAAs (non-alpha poly-amino acids), 2 RiPPs (ribosomally synthesized and post-translationally modified peptides), 1 guanidinotide, 1 sactipeptide, 1 siderophore, and 1 ectoine. Seven putative gene clusters showed high similarity (>70% of genes showed similarity) to ectoine, Ery, geosmin, citrulassin B, mirubactin, nocardicin A, and TLN-05220 gene clusters. The presence of one putative ectoine biosynthetic gene cluster consisted of high levels of saline environment where YIM 96748^T^ was isolated. A high concentration of compatible solute ectoine, which serves as a protective osmolyte, has been found in many halophilic microorganisms (Pastor et al., 2010). Seven putative gene clusters showed moderate similarity (30–70% of genes showed similarity) to nystatin A1, salinichelins, ochronotic pigment, landepoxcin, fortimicin, WS9326, and diazaquinomycin gene clusters. The existence of these putative gene clusters indicated that YIM 96748^T^ offers the opportunity to produce these important antibiotics or their analogs. Twenty-two putative gene clusters showed low similarity (<30% of genes showed similarity) to reported scleric acid, xiamycin, allylmalonyl-CoA, atratumycin, arginomycin, xiamycin, rifamorpholine, ML-449, staphylobactin, echinomycin, BE-43547, arginomycin, teicoplanin, chlorizidine A, chejuenolide, armeniaspirol, rubrolone, phosphonoglycans, rifamorpholine, A54145, and kijanimicin gene clusters. Fifteen putative gene clusters were not conserved relative to any known cluster. The existence of these cryptic secondary metabolite biosynthetic gene clusters implied that YIM 96748^T^ could be a potential source for novel antibiotic discovery. Therefore, we fermented it in 15 different media and found two new compounds and five known ones.

### Compounds Produced by Strain YIM 96748^T^

Compounds 1/2, a yellowish solid, were isolated as a mixture, which was confirmed by analytical HPLC (Supplementary Figure 3). However, we failed to purify compounds 1 and 2 due to the small amount. The same molecular formula for compounds 1 and 2 was assigned as C_11_H_4_O_4_ by interpretation of positive HRESIMS (*m/z* 233.0783 [M + Na]^+^, calculated for 233.0784) (Supplementary Figure 4), indicating 5 degrees of unsaturation. Analysis of the NMR spectra (Supplementary Figure 5) suggested that compounds 1 and 2 shared similar chemical structures. The NMR data were almost identical for compounds 1 and 2, except that the chemical shift values for the C-7, C-8, and C-9 in compound 1 (δ_H_/δ_C_ = 2.70/38.8, 3.94/70.3, and 4.03, 3.92/67.3) were different from those in compound 2 (δ_H_/δ_C_ = 2.77/35.5, 5.00/76.0, and 3.61, 3.53/62.1) (Table 4).

**TABLE 4:** ^1^H (400 MHz) and ^13^C (100 MHz) NMR data for amycoletates A (1) and B (2) in MeOD.

Position	1		2	
	δ_C_, type	δ_H_ (*J* in Hz)	δ_C_, type	δ_*H*_ (*J* in Hz)
1	128.7, C		128.0, C	
2/6	130.0, CH	7.04, d (8.4)	130.0, CH	7.03, d (8.4)
3/5	114.8, CH	6.71, d (8.4)	114.8, CH	6.70, d (8.4)
4	155.5, C		155.5, C	
7	38.8, CH_2_	2.70, d (6.4)	35.5, CH_2_	2.77, dd (9.2, 6.4)
8	70.3, CH	3.94, m	76.0, CH	5.00, m
9	67.3, CH_2_	4.03, dd (9.2, 1.6) 3.92, overlapped	62.1, CH_2_	3.61, dd (12.0, 3.6) 3.53, dd (12.0, 6.0)
10	171.6, C		171.3, C	
11	19.4, CH_3_	2.05, s	19.7, CH_3_	2.00, s

**FIGURE 4:**
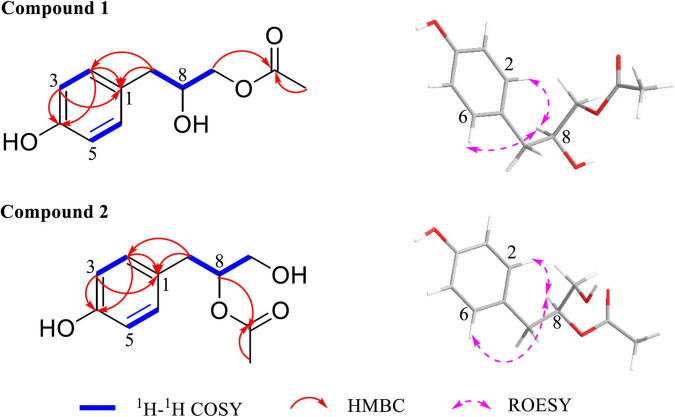
Key ^1^H–^1^H correlated spectroscopy (COSY), heteronuclear multiple bond correlation (HMBC), and rotating-frame Overhauser effect spectroscopy (ROESY) correlations of amycoletates A (1) and B (2).

**FIGURE 5:**
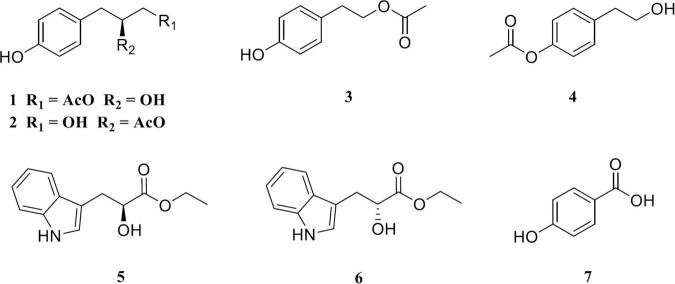
Structures of the compounds from *Amycolatopsis aidingensis* YIM96748^T^.

Then, the structure of compound 1 was ascertained with 2D NMR experiments (Supplementary Figure 5). The ^1^H–^1^H correlated spectroscopy (COSY) correlations of H-2/H-3 and H-5/H-6 and the heteronuclear multiple bond correlations (HMBCs) from H-2, H-3, H-5, and H-6 to C-1 and C-4 indicated the presence of a 1,4-disubstituted benzene in compound 1 (Figure 4). The ^1^H–^1^H COSY correlations of H-7/H-8/H-9 and the HMBCs from H-7 to C-1, C-2, and C-6, together with the chemical shift values of C-4, C-8, and C-9, revealed that the *p*-substituted phenyl group was hydroxyphenyl propane-diol. Furthermore, the HMBCs from H-9 and H-11 to C-10 elucidated that the acetyl linked to C-9. Meanwhile, the 2D NMR data of compound 2 were highly similar to those of compound 1, except for the HMBC from H-8 to C-10, which indicated that the acetyl linked to C-8 in compound 2. Consequently, the planar structures of compounds 1 and 2 are established in Figure 4. The relative configurations of compounds 1 and 2 were deduced from the rotating-frame Overhauser effect spectroscopy (ROESY) experiment (Figure 4). The key ROESY correlations of H-8/H-2 and H-8/H-6 in both compounds 1 and 2 proved the H-8 β-orientation. On the basis of the above, the relative configurations of compounds 1 and 2 were deduced to be 8*S* (Figure 5) and named as amycoletates A and B, respectively.

The structures of the other five purified compounds were confirmed as 4-hydroxy phenethyl acetate (compound 3) (Wei et al., 2018), 2-*p*-acetoxyphenylethanol (compound 4) (Singh et al., 2014), (*S*)-ethyl indole-3-lactate (compound 5), (*R*)-ethyl indole-3-lactate (compound 6) (Marinos et al., 1992), and *p*-hydroxybenzoic acid (compound 7) (Tan et al., 2013) by comparing their NMR data with those of previously published ones. Compounds 1 and 2 were probably produced from compound 7 as a precursor through the one-module plyketide synthase (PKS) pathway and unknown enzymes. Each of gene cluster 14 (rifamorpholine, 12% similarity), gene cluster 36 (atratumycin, 18% similarity), and gene cluster 43 (ML-449, 12% similarity) contained a single module of PKS. Which gene cluster is responsible for the biosynthesis of amycoletates A and B is yet to be verified.

The putative siderophore biosynthetic gene cluster from YIM 96748^T^ showed low similarity (12%) to the staphylobactin biosynthetic gene cluster from *S. aureus* NCTC 8325 (Dale et al., 2004). YIM 96748^T^ showed siderophore activity in our previous studies. The presence of a novel siderophore cluster indicated that this strain is likely to produce new siderophores. The absence of a novel siderophore in the compounds we isolated indicated that this gene cluster might be silent under the tested fermentation conditions. Possibly due to the characteristics of halophilic actinomycetes, other gene clusters might be silenced under traditional fermentation conditions. Intensive studies on fermentation condition optimization, heterologous expression, and genetic modification need to be tried in the future.

## Conclusion

The saline soil-derived halotolerant actinobacterial strain YIM 96748^T^ is a novel species of the genus *Amycolatopsis* whose name was proposed as *Amycolatopsis aidingensis* sp. nov., and the type strain is YIM 96748^T^. It could produce two new compounds, amycoletates A and B, and five known compounds: 4-hydroxy phenethyl acetate, 2-*p*-acetoxyphenylethanol, (*S*)-ethyl indole-3-lactate, (*R*)-ethyl indole-3-lactate, and *p*-hydroxybenzoic acid. The complete genome was sequenced and analyzed. Fifty-one putative secondary metabolite gene clusters were found. Many of them were significantly different from known gene clusters, indicating that this strain could produce many novel compounds. One of the gene clusters 14, 36, and 43 might be responsible for the biosynthesis of the new compounds amycoletates A and B through the one-module PKS pathway and other unknown enzymes. A siderophore cluster is likely to produce novel siderophores because of YIM 96748^T^ having shown siderophore activity. The one strain many compounds (OSMAC) approach and genetic modification including heterologous expression are needed to obtain more novel compounds from this talented antibiotic producer. In conclusion, strain YIM 96748^T^ is a promising candidate for the discovery of novel secondary metabolites.

## Data Availability Statement

The datasets presented in this study can be found in online repositories. The names of the repository/repositories and accession number(s) can be found in the article/Supplementary Material.

## Author Contributions

MY, HZ, and S-KT designed the study, carried out the data analysis, and wrote the manuscript. RL, MW, ZR, and YJ carried out the experiments and participated in the data analysis. All authors have read and approved the manuscript.

## Conflict of Interest

The authors declare that the research was conducted in the absence of any commercial or financial relationships that could be construed as a potential conflict of interest.

## Publisher’s Note

All claims expressed in this article are solely those of the authors and do not necessarily represent those of their affiliated organizations, or those of the publisher, the editors and the reviewers. Any product that may be evaluated in this article, or claim that may be made by its manufacturer, is not guaranteed or endorsed by the publisher.

## Footnotes

1
http://eztaxon-e.ezbiocloud.net/

2
http://enve-omics.ce.gatech.edu/aai/
