# Supplementary material for: Amycolatopsis aidingensis sp. nov., a Halotolerant Actinobacterium, Produces New Secondary Metabolites
Source: Front Microbiol. 2021 Dec 6;12:743116. doi: 10.3389/fmicb.2021.743116 (PMC8685341; doi:10.3389/fmicb.2021.743116)
Supplement: Supplementary file 1 [file Data_Sheet_1.docx]

Supplementary Material

*Amycolatopsis aidingensis* sp.nov., a Halotolerant Actinobacterium Produces New Secondary Metabolites

**Rui Li^a, 1^, Meng Wang ^a, 1^, Zhen Ren^b, 1^, Yang Ji, Min Yin ^a, *^, Hao Zhou ^a, *^ and Shukun Tang ^a, *^**

*Correspondence: Min Yin: yinmin@ynu.edu.cn; Hao Zhou: haozhou@ynu.edu.cn and Shukun Tang: tangshukun@ynu.edu.cn

*Streptomyces avermitilis* MA-4680^T^(GCA_000009765.2)

*Amycolatopsis jiangsuensis* KLBMP 1262^T^(GCA_014204865.1)

*Amycolatopsis anabasis* EGI 650086^T^(GCA_009765355.1)

*Amycolatopsis nigrescens* CSC17Ta-90^T^(GCA_000384315.1)

*Amycolatopsis Antarctica* AU-GU^T^(GCA_002262875.1)

*Amycolatopsis palatopharyngis*1Bdz^T^(GCA_003385185.1)

*Amycolatopsis marina* Ms392A^T^(GCA_900111885.1)

*Amycolatopsis cihanbeyliensis* BNT52^T^(GCA_006715045.1)

***Amycolatopsis aidingensis*YIM 96748^T^**

*Amycolatopsis albispora* WP1^T^(GCA_003312875.1)

*Amycolatopsis magusensis* KT2025^T^(GCA_017875555.1)

*Amycolatopsis suaedae* 8-3EHSu^T^(GCA_004214935.1)

100

100

100

100

100

98

100

100

100

0.050

**Figure S1** Phylogenomics tree showing the phylogenetic relationships between strain YIM 96748^T^ and related members of the genus *Amycolatopsis*. *Streptomyces avermitilis* MA-4680^T^(GCA_000009765.2) was used as the outgroup. Bootstrap values (> 50%) based on 100 resamplings are given at the nodes. Bar, 0.05 substitutions per nucleotide position.


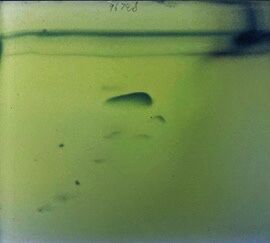


L

DPG

PME

AL

PE

PI

PL

**Figure S2** The main lipid profile of strain YIM 96748^T^.

Abbreviations: DPG, diphosphatidylglycerol; L, unidentified polar lipids; PI, phosphatidylinositol; PE, phosphatidylethanolamine; PL, unidentified phospholipids; AL, unidentified aminolipid; PME, phosphatidylmonomethylethanolamine

**1**

**2**

**Figure S3** HPLC separation profile of **1**/**2**.

(HPLC parameters: stationary phase: Agilent Eclipse XDB-C_18_ 5 µm, 4.6 × 250 mm; mobile phase: H_2_O:methanol = 70:30; temp: 30 °C; flow: 1 mL/min; injection volume: 10 µL; sample concentration: 1 mg/mL in methanol.)


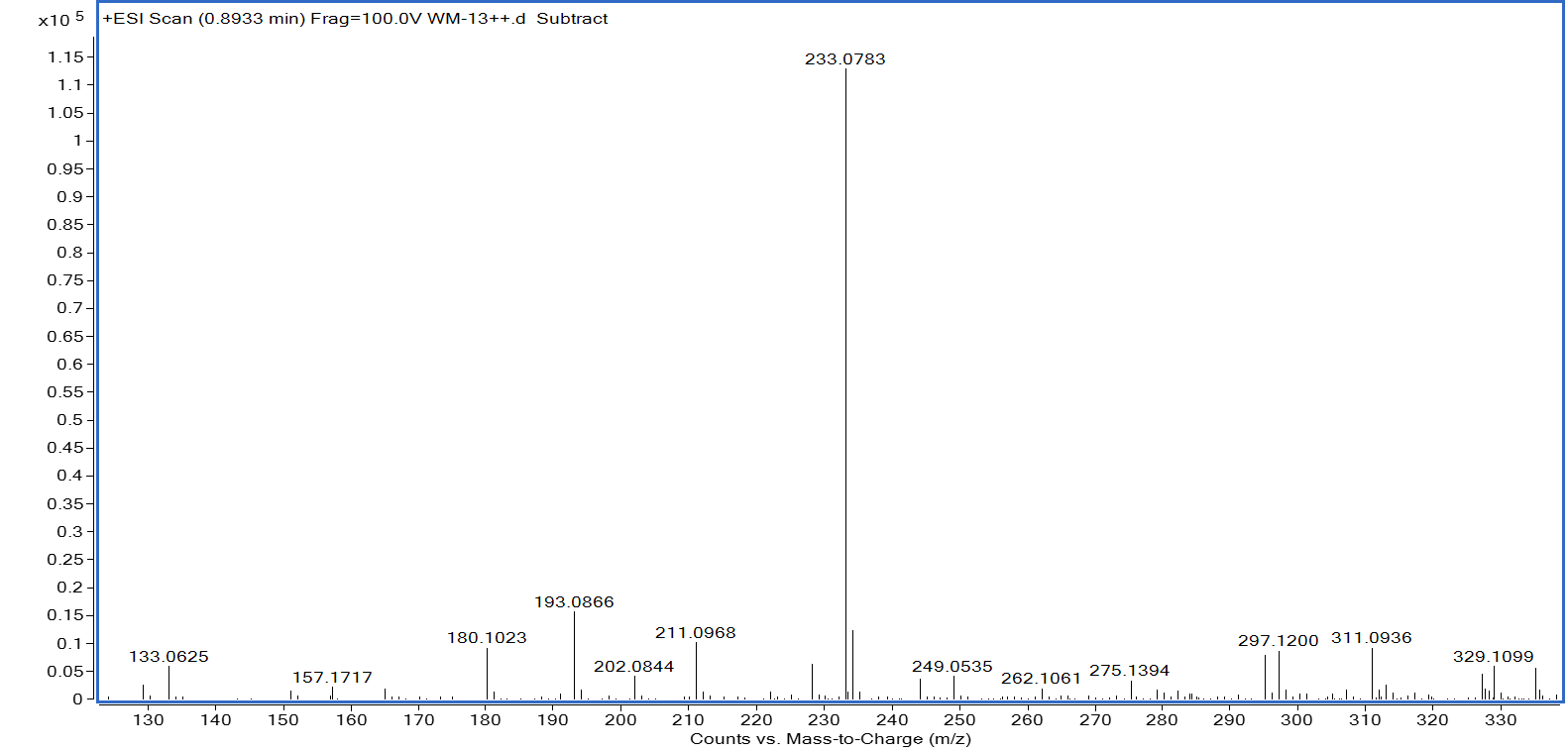


**Figure S4** (+)-HRESIMS data of amycoletates A (**1**) and B (**2**).

(A) ^1^H


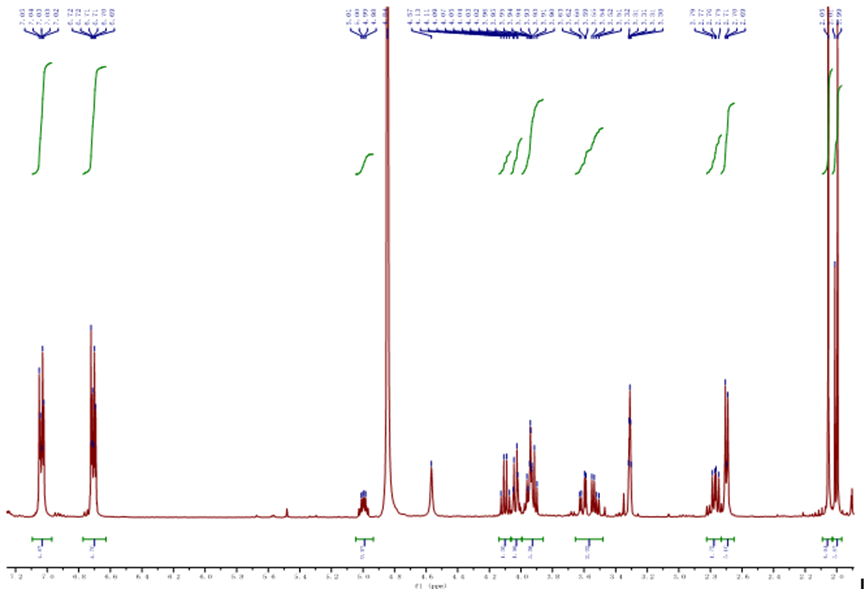


(B) ^13^C & DEPT


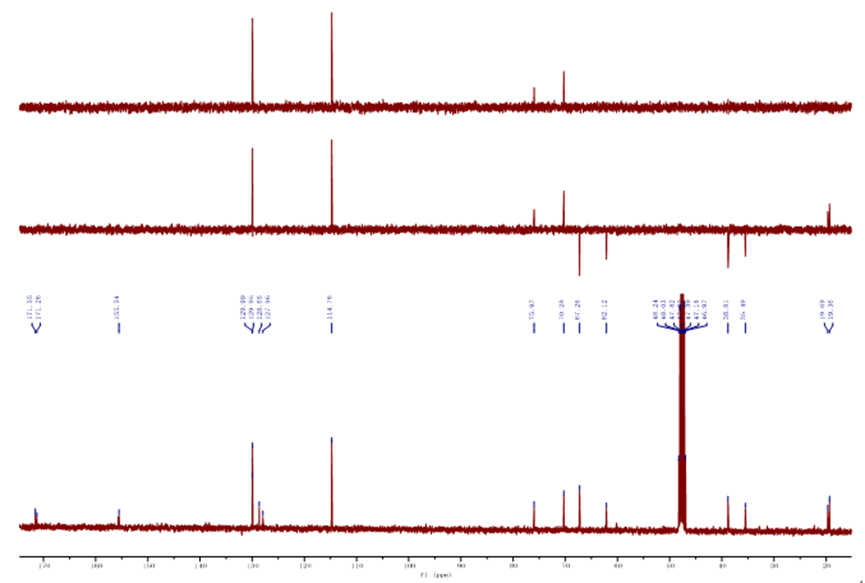


(C) ^1^H-^1^H COSY


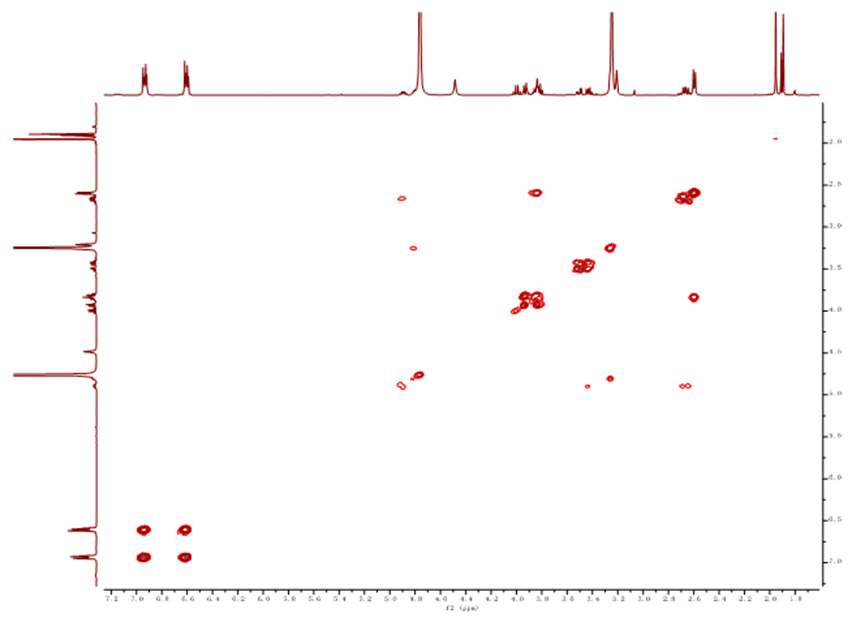


(D) HSQC


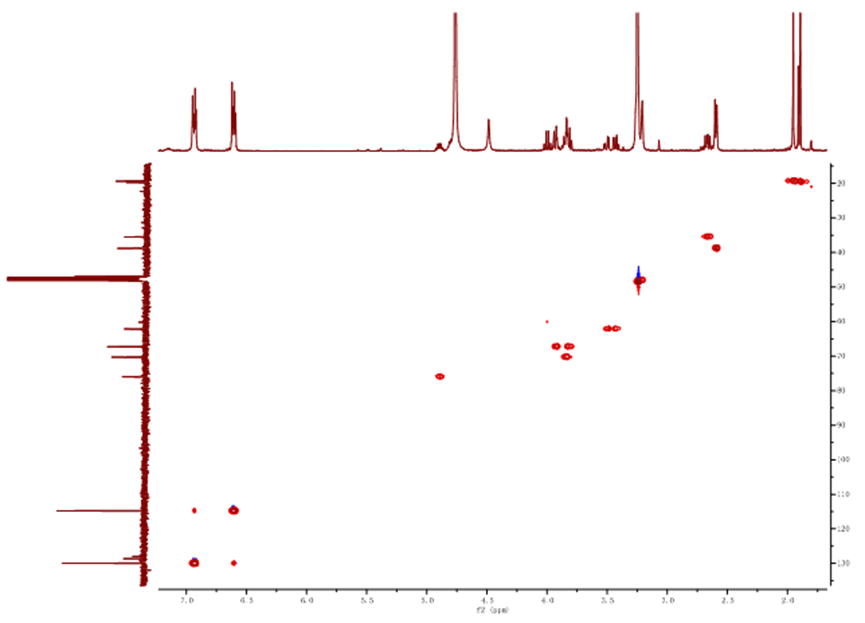


(E) HMBC

**
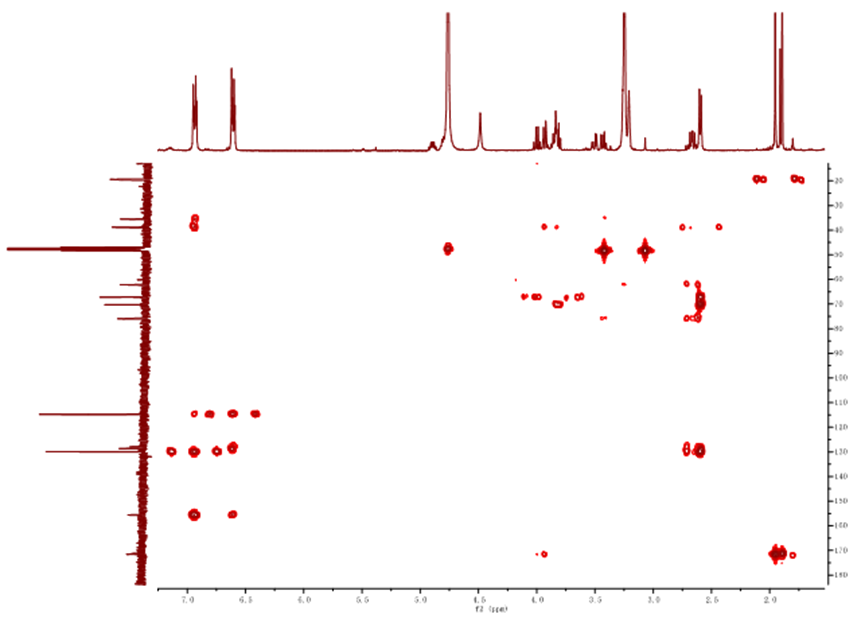
**

(F) ROESY

**
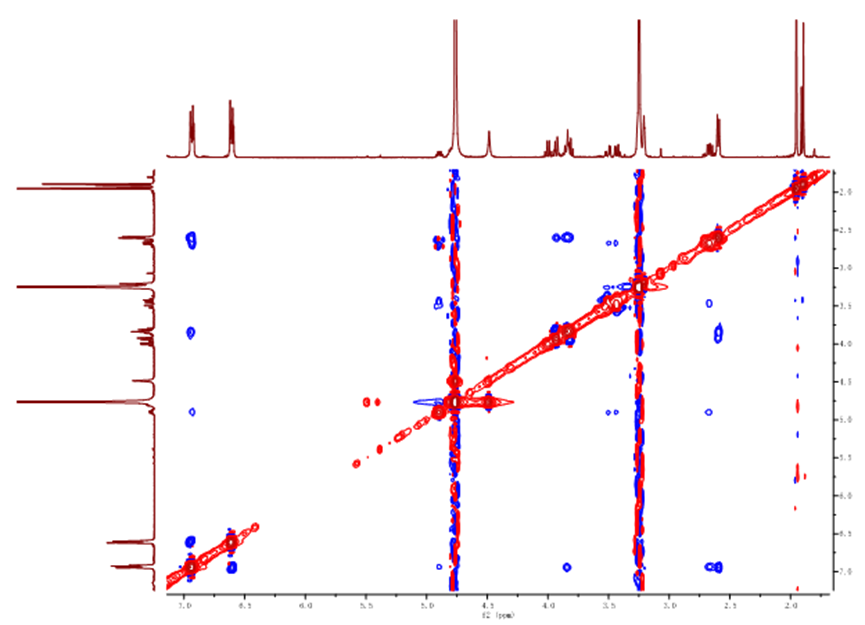
**

**Figure S5** NMR spectra of amycoletates A (**1**) and B (**2**) in MeOD (400 MHz).

(A) ^1^H, (B) ^13^C & DEPT, (C) ^1^H-^1^H COSY, (D) HSQC, (E) HMBC, and (F) ROESY.


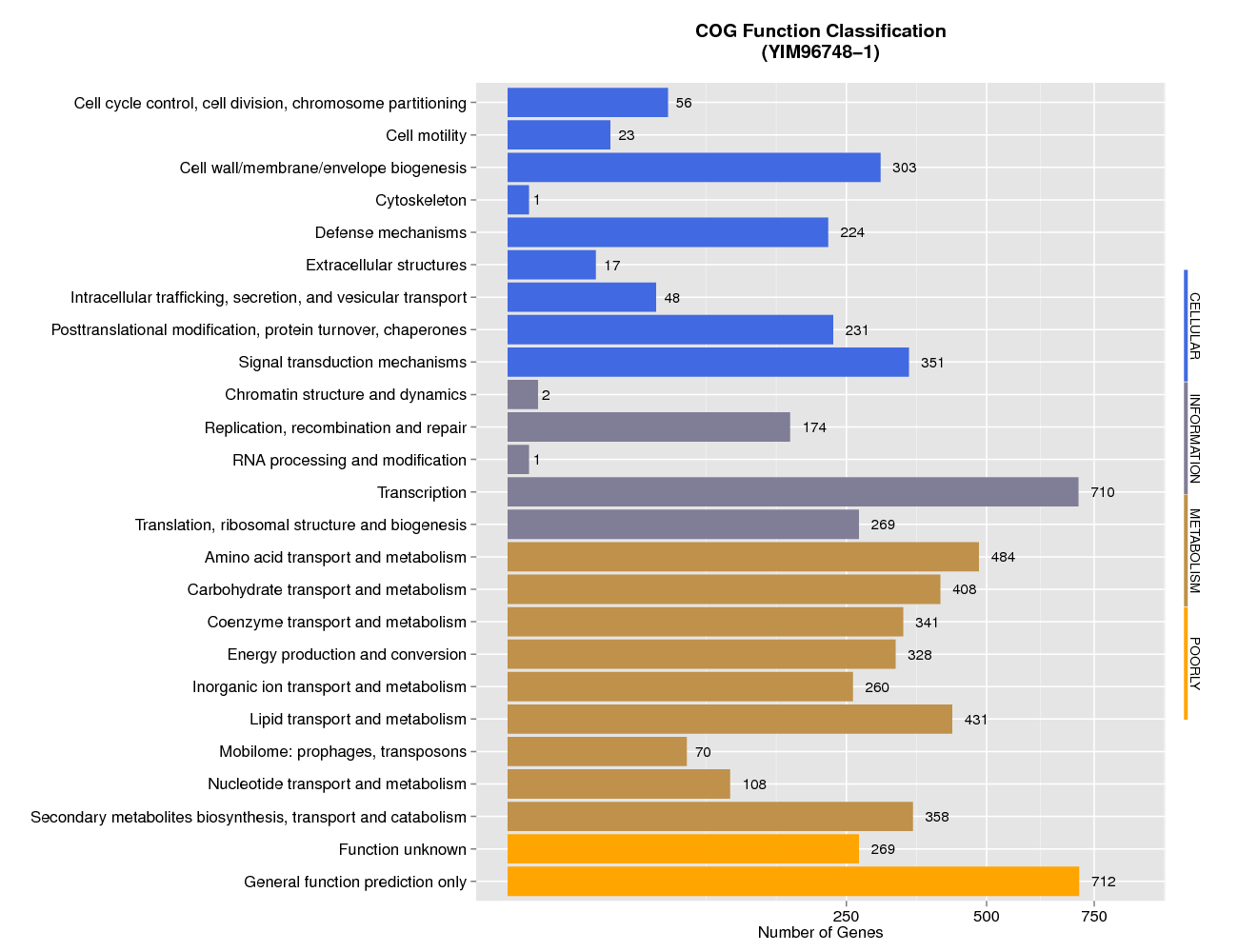


**Figure S6** COG-based functional classification of genes located on YIM 96748 chromosome.


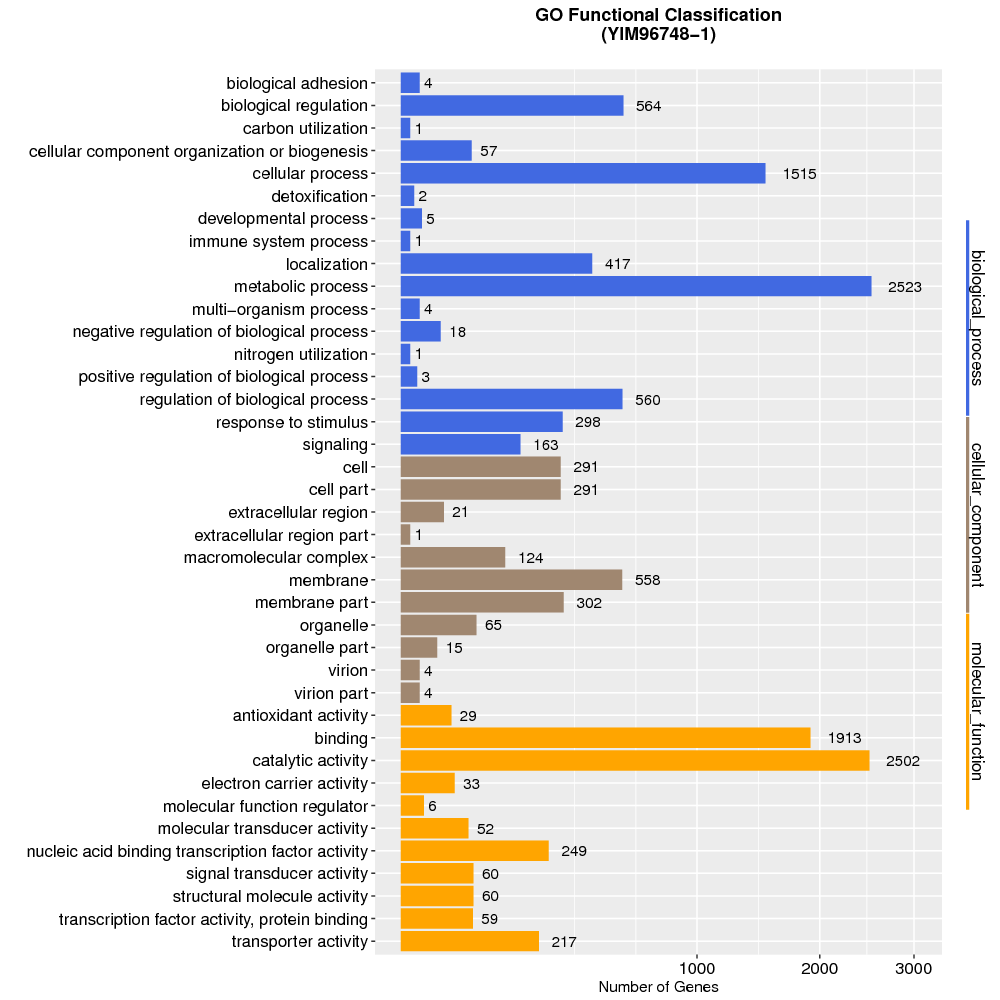


**Figure S7** GO-based functional classification of genes located on YIM 96748 chromosome.


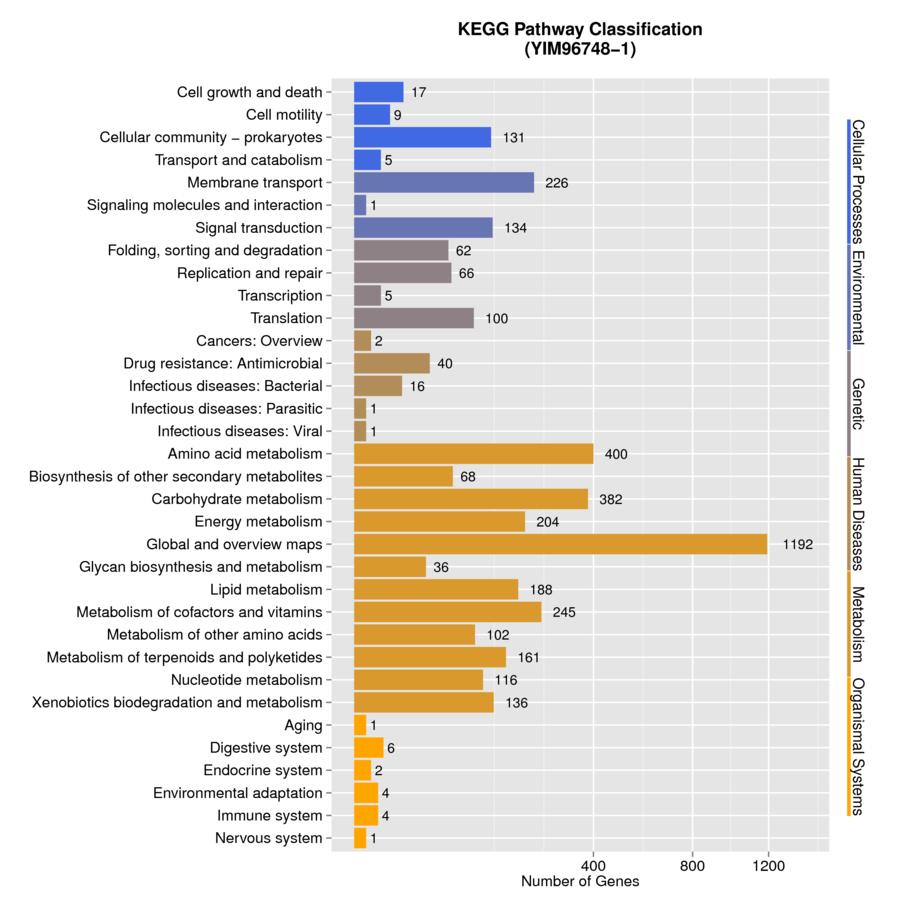


**Fig. S8** KEGG-based functional classification of genes located on YIM 96748 chromosome.


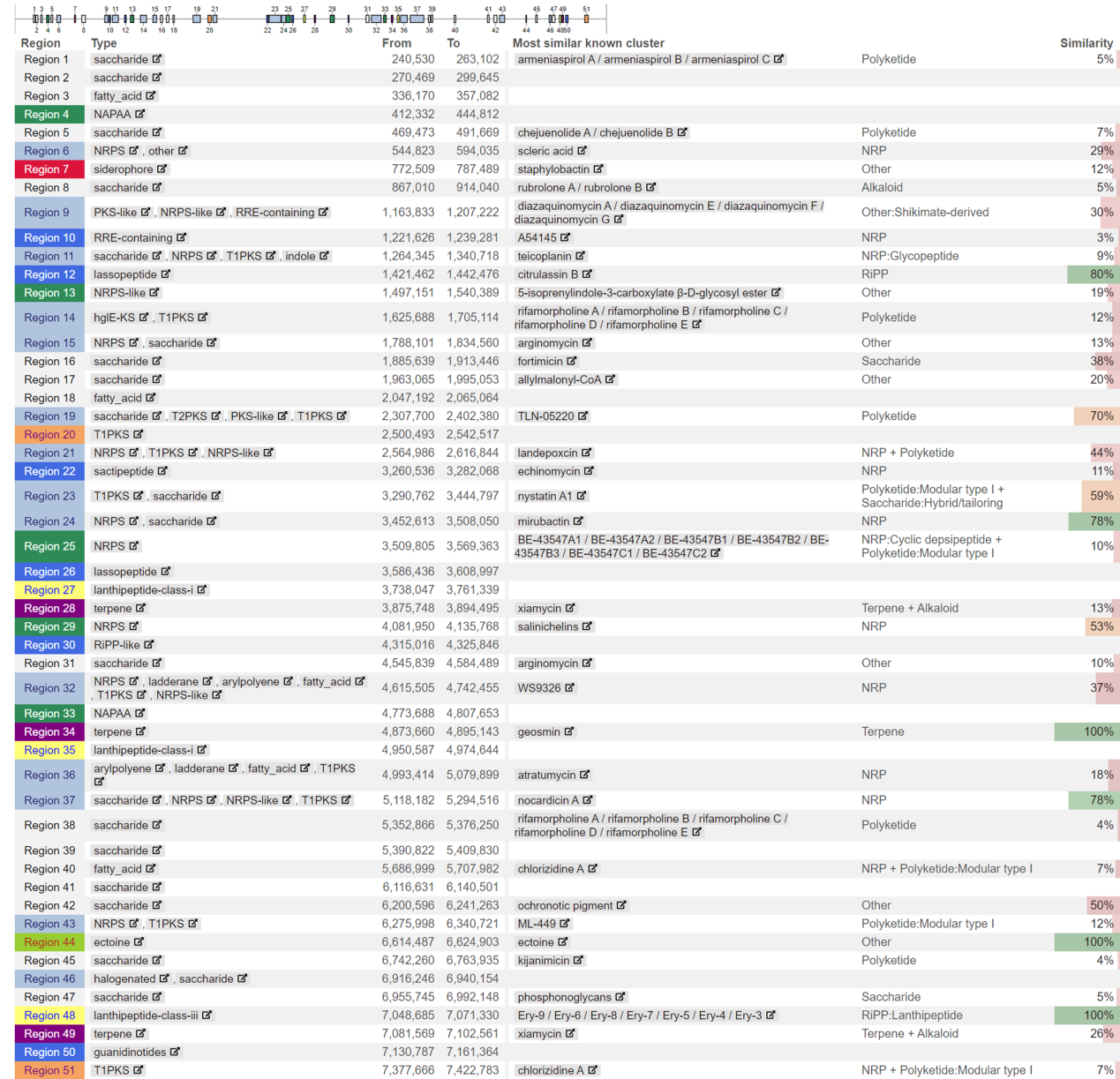


**Figure S9** putative biosynthetic gene clusters.
